# ABC Transporter Subfamily D: Distinct Differences in Behavior between ABCD1–3 and ABCD4 in Subcellular Localization, Function, and Human Disease

**DOI:** 10.1155/2016/6786245

**Published:** 2016-09-28

**Authors:** Kosuke Kawaguchi, Masashi Morita

**Affiliations:** Department of Biological Chemistry, Graduate School of Medicine and Pharmaceutical Sciences, University of Toyama, 2630 Sugitani, Toyama 930-0194, Japan

## Abstract

ATP-binding cassette (ABC) transporters are one of the largest families of membrane-bound proteins and transport a wide variety of substrates across both extra- and intracellular membranes. They play a critical role in maintaining cellular homeostasis. To date, four ABC transporters belonging to subfamily D have been identified. ABCD1–3 and ABCD4 are localized to peroxisomes and lysosomes, respectively. ABCD1 and ABCD2 are involved in the transport of long and very long chain fatty acids (VLCFA) or their CoA-derivatives into peroxisomes with different substrate specificities, while ABCD3 is involved in the transport of branched chain acyl-CoA into peroxisomes. On the other hand, ABCD4 is deduced to take part in the transport of vitamin B_12_ from lysosomes into the cytosol. It is well known that the dysfunction of ABCD1 results in X-linked adrenoleukodystrophy, a severe neurodegenerative disease. Recently, it is reported that ABCD3 and ABCD4 are responsible for hepatosplenomegaly and vitamin B_12_ deficiency, respectively. In this review, the targeting mechanism and physiological functions of the ABCD transporters are summarized along with the related disease.

## 1. Introduction

There are 48 ATP-binding cassette (ABC) transporters in humans that are classified into seven subfamilies, A to G, based on structural organization and amino acid homology [1, 2]. ABC transporters exist on plasma membranes and intracellular compartments such as the mitochondria, peroxisomes, endoplasmic reticulum (ER), Golgi apparatus, and lysosomes and play important roles in transporting a wide variety of substrates across membranes in order to sustain cellular homeostasis. Defects in their functions are related to various diseases [3].

**Figure 1:**
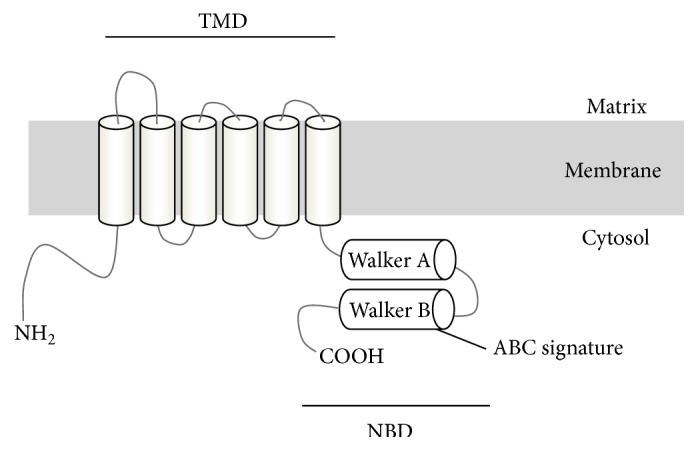
Hypothesized structure of the ABCD transporter. The ABCD transporters are comprised of a half-size ABC transporter, with one transmembrane domain (TMD) and one nucleotide-binding domain (NBD). Six transmembrane domains are located in the NH_2_-terminal half of the transporter, and Walker A, Walker B, and ABC signature are located in the COOH-terminal half of the transporter.

To date, four ABCD transporters have been identified: adrenoleukodystrophy protein (ALDP/ABCD1), ALDP-related protein (ALDRP/ABCD2), the 70-kDa peroxisomal membrane protein (PMP70/ABCD3), and the PMP70-related protein (P70R/ABCD4) [4–7]. The ABCD transporters have a predicted ABC half-transporter structure with one transmembrane domain (TMD) and one nucleotide-binding domain (NBD) (Figure 1). ABCD1–3 are known to be peroxisomal proteins and predominantly function as a homodimer [8], but a heterodimeric structure has also been suggested [9]. It is reported that ABCD1 and ABCD2 are involved in the transport of long and very long chain fatty acids (VLCFA) or their CoA-derivatives into peroxisomes [10, 11]. ABCD3 is thought to play an important role in the transport of branched chain acyl-CoA and the bile acid intermediates di- and tri-hydroxycholestanoyl-CoA (DHCA and THCA) [12]. It is known that ABCD1 and ABCD3 defects are the cause of X-linked adrenoleukodystrophy (X-ALD), a neurodegenerative disease, and hepatosplenomegaly, a liver disease, respectively [5, 13].

ABCD4 was identified by a homology search for ABCD1- and ABCD3-related sequences in a database of expressed sequence tags and is thus considered to be a peroxisomal protein [7]. We reported that ABCD4 is not a peroxisome resident protein but rather an ER resident protein. However, the function of ABCD4 still remained unknown at that time [14]. In 2012, it was reported that mutations in* ABCD4* cause a newly identified inborn error of vitamin B_12_ (cobalamin) metabolism [15]. The dysfunction of ABCD4 results in a failure in lysosomal cobalamin release, which mimics the cobalamin deficiency caused by the defect of lysosomal membrane protein LMBD1 [16]. It is indicated that ABCD4 has a function on the lysosomal membrane. Recently, we showed that ABCD4 is translocated from the ER to lysosomes through an interaction with LMBD1 [17].

As mentioned above, dysfunction of any of the ABCD transporters other than ABCD2 may be the cause of disease. In this review, we will focus on the targeting mechanism of ABCD transporters to peroxisomes or lysosomes, their functions, and related diseases.

## 2. Role of Human Peroxisomes and Lysosomes

### 2.1. Peroxisomes

Peroxisomes are ubiquitous organelles in all eukaryotic cells and participate in various activities related to cellular homeostasis, especially lipid metabolism [18]. The indispensable role of the peroxisome in human health and development is evidenced by the large number of genetic diseases termed peroxisomal disorders [19].

The peroxisomes contain more than 50 different enzymes that catalyze a variety of metabolic pathways, including fatty acid *β*-oxidation, the biosynthesis of ether phospholipids and bile acids, and the metabolism of reactive oxygen species. In addition, peroxisomes fulfill essential roles in the synthesis of the ether phospholipid such as plasmalogen and the *α*-oxidation of phytanic acid. Among these various functions, the *β*-oxidation of fatty acid is generally considered to be the main one [20].

In peroxisomes, *β*-oxidation is performed on VLCFA (>C22:0), branched chain fatty acids (e.g., pristanic acid), bile acid intermediates such as DHCA and THCA [21], poly-unsaturated fatty acid (e.g., tetracosahexaenoic acid), long chain dicarboxylic acids [22], 2-hydroxy fatty acids, and a number of prostanoids. The *β*-oxidation of these substrates is restricted to peroxisomes and does not occur in mitochondria, where long chain, medium chain, and short chain fatty acids (C18 and shorter) are *β*-oxidized.

These fatty acids are imported into peroxisomes via specific transporters as mentioned above, where they are degraded by four *β*-oxidation cycles. Like the activity of mitochondrial *β*-oxidation, the *β*-oxidation performed in peroxisomes is conducted by oxidation, hydration, dehydrogenation, and thiolytic cleavage [23]. In the first step, the acyl-CoA oxidase ACOX1 or ACOX2 catalyze the reaction. ACOX1 preferentially degrades saturated and unsaturated straight chain fatty acids, while ACOX2 has a high affinity for 2-methyl branched fatty acids, such as pristanoyl-CoA, DHCA-CoA, and THCA-CoA. In the second and third steps, D- and L-bifunctional enzymes function as an enoyl-CoA hydratase and a 3-hydroxyacyl CoA dehydrogenase, respectively. Two thiolases, 3-ketoacyl-CoA-thiolase 1 (pTH1) and SCPx (pTH2), are involved in the last step. pTH1 metabolizes only straight chain fatty acids. The branched chain fatty acids and bile acid precursors (pristanic acid, DHCA, and THCA) are solely cleaved by pTH2. Once the fatty acid chains are shortened to medium chain fatty acyl-CoA via peroxisomal *β*-oxidation, they are conjugated to carnitine, exit the peroxisomes, and undergo further *β*-oxidation in mitochondria. On the other hand, choloyl-CoA and deoxycholoyl-CoA are converted to taurine- or glycine-conjugated cholic acid, or deoxycholic acid, by bile acids-CoA: amino acid N-acyltransferase, and then exported into the cytosol.

When functional peroxisomes or enzymes are absent, the undegraded molecules such as VLCFA, DHCA, THCA, and phytanic acid accumulate in the cell, while physiologically essential molecules, such as bile acids and plasmalogen become deficient. In addition to lipid metabolism, peroxisomes play a role in several nonlipid metabolic pathways, including purine, polyamine, glyoxylate, and amino acid metabolism.

Recently, new functions for peroxisome have been reportedly demonstrated. It is reported that peroxisomes as well as mitochondria act as intracellular signaling platforms in innate immunity and are important for early stage antiviral signaling [24]. Moreover, it has been shown that peroxisomes have a role in the transport of free cholesterol from lysosomes to the ER through lysosome-peroxisome membrane contact [25]. It is thus evident that peroxisomes are multifunctional organelles that interact with other organelles such as mitochondria, lysosomes, the ER, and lipid droplets while performing a diverse array of biological functions.

### 2.2. Lysosomes

Lysosomes are membrane-bound intracellular organelles present in all eukaryotic cells that internally have an acidic pH (~5.0) that is maintained by an ATP-dependent proton pump, the V-type H^+^-ATPase. Lysosomes are dynamic organelles crucially involved in many physiological processes, such as the degradation and/or recycling of macromolecules, cholesterol homeostasis, plasma membrane repair, pathogen defense, cell death, and cell signaling, as well as being core regulators of cell homeostasis [26]. Two classes of proteins are essential for these lysosomal activities. One is comprised of soluble lysosomal hydrolases referred to as acid hydrolases. Acid hydrolases function at a low pH in this organelle and possess a wide variety of substrate specificities. More than 60 hydrolases (including proteases, peptidases, phosphatases, nucleases, glycosidases, sulfatases, and lipases) have been identified to date [27]. The other class is made up of integral lysosomal membrane proteins. These have a variety of functions, including the transport of substrate and metabolic products, establishment of the pH gradients, vesicular transport, and maintenance of lysosomal structural integrity. There are 25 lysosomal membrane proteins identified thus far, but the existence of additional membrane proteins is expected [28–30].

The primary cellular function of the lysosome is the degradation and recycling of macromolecules. During these processes, cytoplasmic components are delivered to lysosomes by endocytosis, phagocytosis, or autophagy. Several classes of macromolecules are hydrolyzed by soluble lysosomal hydrolases, including proteins, polysaccharides, lipids, and nucleic acids. The end products of lysosomal digestion are recycled in the cell after diffusion or undergo carrier-mediated transport across the lysosomal membrane. The process of autophagy is classified into macroautophagy, microautophagy, and chaperone-mediated autophagy [31]. Macroautophagy is a process whereby cytoplasmic organelles and substances are sequestered within autophagosomes that in turn fuse with lysosomes and their contents are degraded. Microautophagy is the direct sequestration of cytoplasmic cargo at the boundary of the lysosomal membrane. During chaperone-mediated autophagy, cytosolic proteins possessing specific recognition motifs are delivered to lysosomes via a chaperone in a lysosomal receptor LAMP-2A dependent manner.

Cholesterol is an essential structural element of cellular membranes as well as a precursor for the synthesis of steroid hormones, bile acids, and lipoproteins. Cellular cholesterol homeostasis is controlled by lysosomal cholesterol efflux through the Niemann-Pick type C1 (NPC1) and NPC2 proteins [32, 33]. NPC1 is a large protein with 13 transmembrane domains which localizes to the membranes of endosomes and lysosomes. NPC2 exists in endosomes and lysosomes as a soluble protein.

Lysosomes are also involved in a secretory pathway known as “lysosomal exocytosis” that plays a major role in important processes such as the immune response, cell signaling, and plasma membrane repair. Plasma membrane injury is a common event in mammalian cells, especially in cells that operate under conditions of mechanical stress. Plasma membrane damage results in calcium influx, after which lysosomal exocytosis and plasma membrane repair are initiated by calcium binding to synaptotagmin 7 at the lysosomal membrane [34]. Lysosomal cell death signaling is triggered by a release of lysosomal cathepsins through an as yet unknown membrane protein.

The lysosomal system is of considerable biomedical importance. Lysosomal dysfunction causes and contributes to numerous diseases, the majority of which are classified as lysosomal storage diseases (LSDs). LSDs result from defects in soluble lysosomal hydrolases or lysosomal membrane proteins. Over 50 LSDs have been identified to date [30].

## 3. Targeting of ABCD Transporters to the Peroxisome or Lysosome

The subcellular localization of proteins is strictly controlled in cells, as it is inherently important for their vital functioning. For the trafficking of ABCD transporters, an NH_2_-terminal hydrophilic region containing an H0 motif plays an important role [35]. The H0 motif is a hydrophobic segment adjacent to the NH_2_-terminal portion of TMD1.

**Figure 2:**
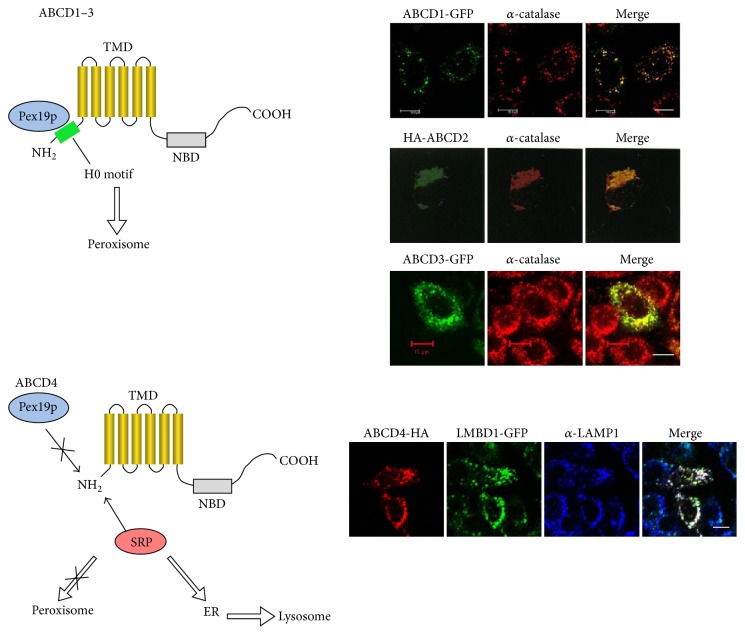
Targeting and localization of the ABCD transporters. For the trafficking of the ABCD transporters, an NH_2_-terminal H0 motif plays an important role. The ABCD1–3 forms possessing this H0 motif are selectively captured by Pex19p and destined for peroxisomes. In contrast, ABCD4 hardly interacts with Pex19p because of its lack of the H0 motif, and, as a result, ABCD4 is recognized by a signal recognition particle (SRP) and integrated into the ER membrane. Subsequently, ABCD4 is translocated to lysosomes through the association with LMBD1. GFP-tagged ABCD1 and ABCD3 were expressed in CHO cells. The distribution of ABCD1 and ABCD3 was compared with that of peroxisomes stained with anti-catalase [36]. The localization of ABCD2 was cited from [6] with some modification. HA-tagged ABCD2 was expressed in COS cells. The distribution of HA-ABCD2 was compared with that of catalase. HA-tagged ABCD4 was expressed in CHO cells stably expressing LMBD1-GFP [17]. The subcellular localization of ABCD4-HA and LMBD1-GFP was compared with that of lysosomes labeled with anti-LAMP1. Bar, 10 *μ*m.

The ABCD transporters are translated on free polysomes. The ABCD1–3 forms possessing the H0 motif are selectively captured by Pex19p, which is essential for the early steps of peroxisome biogenesis and most likely also involved in peroxisomal membrane synthesis and then destined for peroxisomes [8]. In contrast, ABCD4 hardly interacts with Pex19p because of its lack of the NH_2_-terminal H0 motif, and as a result, ABCD4 is recognized by certain signal recognition particles and integrated into the ER membrane [14]. Subsequently, ABCD4 is translocated to lysosomes (Figure 2) [17].

Concerning the trafficking of the newly synthesized peroxisomal ABCD transporters, ABCD3 has been studied in greatest detail [36–39]. As ABCD3 is a hydrophobic integral membrane protein, binding with Pex19p is indispensable for ABCD3 to retain its soluble form and proper conformation for targeting to peroxisomes. In order to investigate the region of ABCD3 (AA.1–659) that is critical for targeting to peroxisomes, Kashiwayama et al. prepared various truncated or mutated ABCD3 fused with GFP. The COOH terminally truncated ABCD3 (AA.1–144)-GFP containing deduced TMD1 and TMD2, along with GFP-ABCD3 (AA.263–375) containing TMD5 and TMD6, was found to be localized to peroxisomes. Further analysis using mutated ABCD3 revealed that ABCD3 is recognized and binds to Pex19p at the NH_2_-terminal hydrophobic motif constituted by Leu^21^-Leu^22^-Leu^23^ and the region of TMD5-TMD6. Subsequently, they prepared various mutated forms of ABCD3 to identify the peroxisomal membrane protein targeting signals (mPTSs). It was suggested that the hydrophobic amino acid pairs Ile^70^-Leu^71^ and Ile^307^-Leu^308^ adjacent to TMD1 and TMD5, respectively, are essential for the targeting to peroxisomes. Thus, ABCD3 forms a complex with Pex19p that is transported to peroxisomes by mPTSs. Finally, ABCD3 is inserted into the peroxisomal membranes through a putative proteinaceous receptor on the peroxisomal membrane. In this process, at least two TMDs (TMD1 and TMD2) are required for proper insertion. Furthermore, it was shown that the TMD1 segment of ABCD3 possesses a potent ER targeting function and a certain cis-acting element in order for TMD1 to suppress ER targeting [40]. For this suppression, certain NH_2_-terminal nine amino acids, especially Ser^5^, are indispensable.

The targeting of ABCD1 to peroxisomes has also been characterized [41]. It was shown that the 14-amino-acid motif (F(F/L)X(R/Q/K)(L/F)(L/I/K)XLLKIL(F/I/V)P) adjacent to TMD1 functions as an mPTS of ABCD1, and it was demonstrated that the substitution or deletion of these hydrophobic residues significantly reduced the targeting efficiency. In particular, the three amino acids Leu^78^-Leu^79^-Arg^80^ were shown to be critical for peroxisomal targeting of ABCD1. This region corresponds to Ile^70^-Leu^71^-Lys^72^ in ABCD3, which was identified as an mPTS in ABCD3. In ABCD2, a potential Pex19p binding site was also identified as ABCD1 [41]. This corresponds to AA.84–97, which are localized in proximity to the putative TMD1. However, no experimental data are available as yet to support the functionality of this putative Pex19p binding site.

Newly synthesized ABCD4 is inserted into ER membranes and then translocated to lysosomes through an interaction with the lysosomal membrane protein LMBD1. Very recently, we demonstrated the underlying mechanism of this translocation [17]. When ABCD4 is exogenously expressed alone in HuH7 cells, ABCD4 is localized in ER membrane and exists as homodimer. However, coexpression of LMBD1 in the cells drastically changes the localization of ABCD4 to lysosomes. During this translocation, the ABCD4 dimer forms a complex with LMBD1. To identify the regions of ABCD4 critical for the translocation to lysosomes, we prepared chimeric ABCD4 proteins in which each TMD of ABCD4 was exchanged to the corresponding putative transmembrane helix with ABCD1, which does not interact with LMBD1. The distribution pattern of the ABCD4 chimeras 1–6 coexpressed with LMBD1 revealed that the regions around transmembrane helices 2 and 5 of ABCD4 are critically important for the translocation of ABCD4 to lysosomes along with LMBD1. As mentioned above, wild-type ABCD4 exists as a homodimer on the ER membrane. However, the ABCD4 chimeras 2 and 5 exist as a bigger complex, indicating higher-order oligomer formation, and this impaired ABCD4 dimer formation disturbs the targeting to lysosomes with LMBD1. Concerning the targeting of LMBD1 to lysosomes, LMBD1 possesses a putative AP-2 binding motif [42]. Mutant LMBD1, with this motif modified, does not localize to lysosomes but to the cell surface. When this mutant LMBD1 and ABCD4 are coexpressed, the distribution pattern of ABCD4 is superimposable on the mutant LMBD1 on the plasma membrane of the cells. Hence, the translocation of ABCD4 from the ER to lysosomes depends on the lysosomal targeting ability of LMBD1. Furthermore, it was confirmed that endogenous ABCD4 was localized to both lysosomes and ER in HEK293 cells by equilibrium iodixanol density gradient centrifugation. Lysosomal localization of ABCD4 was reduced to ~40% in LMBD1 knockout HEK293 cells. These results show that LMBD1 is responsible for the localization of endogenous ABCD4. The subcellular localization of ABCD4 is determined through its association with an adaptor protein.

## 4. Function of the ABCD Proteins

### 4.1. ABCD1

**Figure 3:**
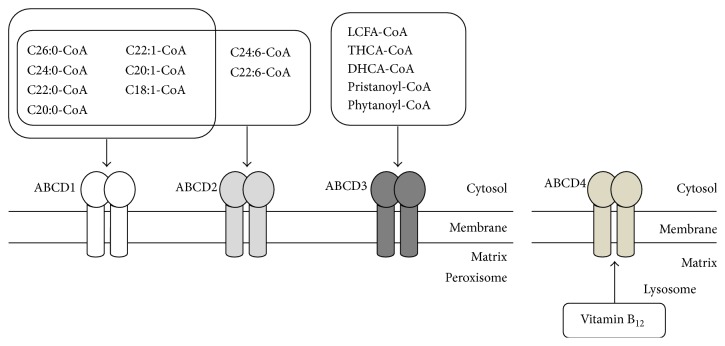
Substrate specificity of the ABCD transporters. ABCD1–3 predominately exist as a homodimer. ABCD1 and ABCD2 have overlapping substrate specificities toward saturated and monounsaturated VLCFA-CoAs. However, ABCD1 has a higher specificity to C24:0-CoA and C26:0-CoA than ABCD2. In contrast, ABCD2 has a higher specificity for polyunsaturated C22:6-CoA and C24:6-CoA. ABCD3 is thought to be involved in the transport of LCFA-CoA, branched chain acyl-CoA, and the bile acid intermediates, THCA-CoA and DHCA-CoA. ABCD4 is deduced to be involved in the transport of cobalamin from lysosomes to the cytosol.

A defect in the ABCD1 protein causes X-ALD, which is characterized by the accumulation of VLCFA in tissues. Thus, it is thought that ABCD1 transports VLCFA across the peroxisomal membrane for *β*-oxidation of VLCFA. This is supported by the result that exogenous expression of ABCD1 in X-ALD skin fibroblasts recovered the VLCFA *β*-oxidation, and, consequently, the VLCFA content was decreased to normal in the fibroblasts [43]. Furthermore, it has been demonstrated that ABCD1 is involved in the transport of saturated, monounsaturated, and polyunsaturated VLCFA-CoA, such as C18:0-, C22:0-, C24:0-, C26:0-, C18:1-, and C24:6-CoA, across the peroxisomal membrane. This was shown by expressing human ABCD1 in the yeast* pxa1/pxa2*Δ mutant, which lacks peroxisomal half-size ABC transporters (Figure 3) [10, 11]. However, the mechanism of fatty acid transport by ABCD1 is controversial. Two models are generally utilized. In the first model, esterified fatty acids are delivered directly to the peroxisomal matrix [44]. In the other model, acyl-CoA is hydrolyzed prior to its entry into the peroxisomal matrix and reesterified by a peroxisomal acyl-CoA synthetase [45]. The first model depends on the fact that *β*-oxidation of VLCFA-CoA directly depends on ABCD1 without the need for any of the additional CoA that is required for reesterification of VLCFA by the acyl-CoA synthetase in the peroxisomes isolated from fibroblasts. On the other hand, it was confirmed that the CoA moiety is cleaved during the transport cycle using isotopic labeling of yeast cells with ^18^O. Furthermore, it was shown that CTS, a homolog of human ABCD1 in* Arabidopsis thaliana*, itself possesses intrinsic acyl-CoA thioesterase activity [46]. We also confirmed that the human ABCD1 expressed in the methylotrophic yeast also hydrolyzes acyl-CoA. However, the precise transport mechanism of VLCFA-CoA as yet is still unclear.

### 4.2. ABCD2

It is known that ABCD2 shares functional redundancy with ABCD1 [47]. In fact, overexpression of ABCD2 in X-ALD fibroblasts fully restores the *β*-oxidation defect. However, the phenotype of fatty acid abnormalities in* Abcd2* ^−/−^ mice is different from that in* Abcd1* ^−/−^ mice [48].* Abcd1* ^−/−^ mice exhibit a higher accumulation of C24:0 and especially C26:0. In contrast,* Abcd2* ^−/−^ mice display fatty acid abnormalities, especially at the level of mono- and polyunsaturated fatty acids, indicating the different substrate specificities of ABCD1 and ABCD2. This was shown in experiments using the yeast* pxa1/pxa2*Δ mutant expressing ABCD1 and/or ABCD2 [11]. ABCD2 shows overlapping substrate specificities with ABCD1 toward saturated and monounsaturated fatty acids. ABCD1 has a higher specificity for saturated VLCFA-CoA. In contrast, ABCD2 has an affinity for polyunsaturated fatty acids such as C22:6-CoA and C24:6-CoA, but ABCD1 does not (Figure 3). These results are consistent with the results from H4IIEC3 cells overexpressing ABCD2 [49]. However, the actual function of ABCD2* in vivo* is still unclear because the endogenous expression level of ABCD2 is quite low in human cells and there is no reported disease caused by a mutation of ABCD2.

### 4.3. ABCD3

ABCD3 is one of the most abundant peroxisomal membrane proteins, at least in hepatocytes [50], and has been reported to be involved in the transport of various fatty acids. It has been confirmed that ABCD3 possesses substrate specificity that overlaps with ABCD1 and ABCD2, but ABCD3 has been shown to have roles in the transport of more hydrophilic substrates, such as long chain, saturated, unsaturated, long-branched chain, and long chain dicarboxylic fatty acids, using the yeast* pxa1/pxa2*Δ mutant expressing human ABCD3 (Figure 3) [12]. In* Abcd3* ^−/−^ mice, there are clearly evident bile acid abnormalities. In the liver, bile, and intestine, there was a significant reduction of chenodeoxycholic acid and cholic acid conjugated with taurine or glycine. On the other hand, a remarkable increase of bile acid intermediates with C27 was observed in the liver, bile, and intestine. Furthermore, for animals on a phytol diet, there was a marked accumulation of phytanic and pristanic acid in the plasma of* Abcd3* ^−/−^ mice [13]. Thus, ABCD3 is involved in the transport of branched chain fatty acids and C27 bile acid intermediates into peroxisomes. Whether ABCD3 possesses intrinsic acyl-CoA thioesterase activity like ABCD1 has yet to be elucidated.

### 4.4. ABCD4

Recently, it was shown that ABCD4 is involved in a newly discovered inherited defect affecting vitamin B_12_ (cobalamin) metabolism [15]. In humans, cobalamin forms a complex with transcobalamin in the blood stream. This complex is taken up into lysosomes by endocytosis. Cobalamin is then released into the cytosol and converted into the two active cofactors methylcobalamin (MeCbl) and adenosylcobalamin (AdoCbl) [51]. A defect in ABCD4 results in the accumulation of cobalamin in lysosomes. In patient fibroblasts, the intracellular enzyme-bound cobalamin level is significantly lower than control fibroblasts. The expression of wild-type ABCD4 in patient fibroblasts was shown to normalize the intracellular enzyme-bound cobalamin level and remarkably increase the synthesis of MeCbl and AdoCbl. On the other hand, the expression of ABCD4 mutated at the putative ATP-binding site leads to a reduced synthesis of both cofactors. This suggests that the ATPase activity of ABCD4 may be involved in the intracellular processing of cobalamin. As mentioned above, mutations of ABCD4 and LMBD1 result in a quite similar phenotype. This suggests that these two proteins function as a complex in the process of transporting cobalamin from lysosomes to the cytosol. Very recently, we revealed that LMBD1 associates with ABCD4 on the ER and supports the translocation of ABCD4 to lysosomes [17]. The bacterial ABC transporter BtuCD is involved in the import of cobalamin into the cytoplasm across the inner membrane and possesses ATPase activity [52]. ABCC1, a multidrug resistance-associated protein on the plasma membranes of mammalian cells is suggested to be involved in the export of cobalamin out of cells [53]. ABCC1 itself also possesses ATPase activity. Therefore, we postulate that ABCD4 is composed of a transporter unit and that LMBD1 is an accessory protein.

ABCD1–3 transport substrates from the cytosol to peroxisomes. In contrast, ABCD4 reportedly transports cobalamin from lysosomes to the cytosol, although it has not yet been demonstrated that ABCD4 directly transports cobalamin. It is of special interest to determine the mechanism underlying the difference in the direction of this transport.

## 5. Mutation and Disease

### 5.1. ABCD1

X-ALD is the most frequent peroxisomal disorder caused by mutations or deletions of the* ABCD1* gene with an average incidence in males of 1 : 20000 [54]. The* ABCD1* gene is composed of 10 exons and encodes the ABCD1 protein with length of 745 amino acids [5]. At present, 717 nonrecurrent mutations are described in the X-ALD database (http://www.x-ald.nl). The mutation types consist of missense (61%), frame shift (22%), nonsense (10%), amino acid insertion/deletion (4%), and one or more exons deleted (3%). Deletion, frame shift, and nonsense mutations generate truncated ABCD1 proteins. In contrast, missense mutations frequently result in the instability and thereby a decrease in the amount of the ABCD1 protein. It seems likely that an ABCD1 protein with a missense mutation undergoes incorrect folding, miss-targeting, and a failure to form a dimer, all of which result in the instability of the mutant ABCD1 protein.

**Figure 4:**
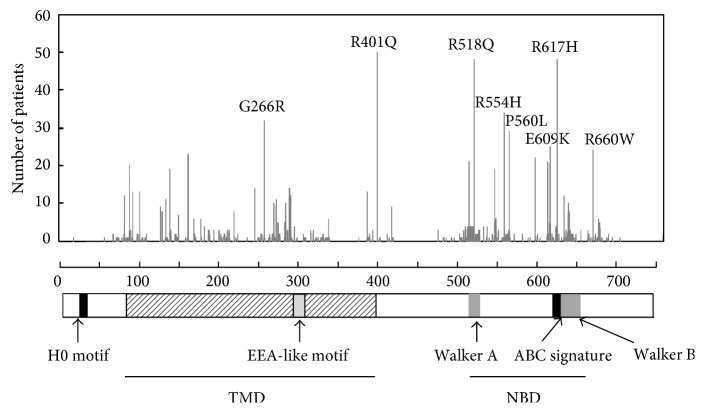
Distribution of the missense mutations reported in X-ALD patients. 209 different amino acids with single amino acid substitutions have been identified. Based on the X-ALD database, G266R, R401Q, R518Q, R554H, P560L, E609K, R617H, and R660W are the most frequent. The mutant ABCD1 proteins, such as G266R, R401Q, and R554H, are unaffected in terms of protein stability. In contrast, mutant ABCD1 proteins with missense mutations (R518Q, P560L, E609K, R617H, and R660W) in COOH-terminal nucleotide-binding domains are decreased or not detected in the corresponding patient fibroblasts.

The missense mutations that occur in X-ALD patients have been found throughout the entire gene and there are 209 different amino acids with single amino acid substitutions that have been identified as disease-causing missense mutations [55]. Based on the X-ALD database, G266R, R401Q, R518Q, R554H, P560L, E609K, R617H, and R660W are the most frequently identified (Figure 4). The mutations G266R (loop 4), R401Q (downstream of TMD6), and R554H (between Walker A and Walker B) did not exert any effect on protein stability, indicating that these amino acid residues are critical for their function. In contrast, mutant ABCD1 proteins with missense mutations (R518Q, P560L, E609K, R617H and R660W) in COOH-terminal nucleotide-binding domains are either decreased or not detected. It is thus likely that the dysfunction of the ABCD1 protein is mainly caused by the instability of the mutant ABCD1 protein. We previously reported that mutant ABCD1 protein with a missense mutation in the COOH-terminal was frequently degraded by proteasomes or additional proteases [56].

ABCD protein is a half-sized ABC transporter that needs to form a dimer for its proper function [57]. The NBD forms a sandwich dimer with two composite ATP-binding sites comprising Walker A, Walker B, and H-loop of one NBD and the signature motif of the other NBD [58]. The missense mutation for R518Q, E609K, and R617H is located in the region of Walker A, the signature motif, and Walker B, respectively, suggesting that these mutant proteins may fail to form dimer. In contrast, it was shown that the 87 COOH-terminal amino acids (AA.658–745) are involved in the dimer formation [57]. In addition, no missense mutation has been found in the COOH-terminal region after AA.693. These results suggest that the COOH-terminal region of AA.658–693, but not AA.694–745, is required for dimerization. Accordingly, X-ALD fibroblasts having truncated ABCD1 protein (AA.1–693) reportedly retain VLCFA *β*-oxidation activity [59]. In X-ALD fibroblasts with a nonsense mutation (Q672X), the translation product lacking the 74 COOH-terminal amino acids (AA.1–671) was not detectable, indicating that AA.672–745 is essential for its correct folding [60]. Taken together, the region between AA.672 and 693 appears to be critically important for dimerization. This is supported by the recent report that showed that the COOH-terminal region of the yeast peroxisomal ABCD protein Pxa2p corresponding to W679 and L684 in ABCD1 is involved in the heterodimerization that occurs with Pxa1p [61].

### 5.2. ABCD3

Recently, a patient with an ABCD3 defect was identified [13]. The patient exhibited hepatosplenomegaly and severe liver disease along with a significant accumulation of the peroxisomal C27 bile acid intermediates DHCA and THCA in plasma. The peroxisomal *β*-oxidation of C26:0 and *β*-oxidation enzyme activities were normal, whereas the *β*-oxidation of pristanic acid was decreased. The* ABCD3* gene is composed of 24 exons, and the normal length of* ABCD3* cDNA is 1980 bp. In this patient, there was a deletion of exon 24 and part of the 3′UTR (c.1903-573_^*∗*^1108). This deletion results in a truncated form of ABCD3 lacking 24 COOH-terminal amino acids, but the truncated ABCD3 is nevertheless still present in the fibroblasts of the patient. As shown in our targeting experiment, in ABCD3, the deletion of the NBD (AA.375–660) does not exert any influence on the sorting to peroxisomes [36]. Therefore, it is assumed that the truncated ABCD3 loses the ability to transport substrates. In the case of ABCD1, it is deduced that the region of AA.672–693 is critically important for dimerization, as described above. The 24 COOH-terminal amino acids of ABCD3 lost in the patient overlap with this region. Hence, it seems that the truncated ABCD3 is unable to form dimer and this is the reason for the lack of functionality.

### 5.3. ABCD4

It was shown that ABCD4 is involved in a newly identified inherited defect affecting cobalamin metabolism [15]. To date, nine inherited defects in the intracellular processing of cobalamin are known, designated cblA to cblG, cblJ, and mut [15, 62]. These defects result in the accumulation of methylmalonic acid, homocysteine, or both, which in turn leads to methylmalonic aciduria and/or isolated homocystinuria. The mutation of* ABCD4*, which is known as the cblJ complementation group, results in the failure of cobalamin release from lysosomes. At present there are four patients with a reported mutation in* ABCD4* gene [15, 63]. Patient 1 possesses two mutations: a missense mutation, c.956A>G (p.Y319C), and a dinucleotide insertion, c.1746_1747insCT (p.E583LfsX9), resulting in a frameshift and the introduction of a premature stop codon that results in the removal of 14 COOH-terminal amino acids. Patient 2 also possesses two mutations: c.542+1G>T and c.1456G>T, resulting in in-frame deletions of p.D143_S181del and p.G443_S485del. Patients 3 and 4 possess a missense mutation, c.423C>G (p.N141K). There are no data on whether mutated ABCD4 is present or not in these patients. In the case of patient 1, N141 is located in the loop between the second and third transmembrane helices of the TMD, based on the predicted topology of the ABCD4. The substitution of this asparagine to lysine is predicted to be “probably damaging” by the PolyPhen-2 program (http://genetics.bwh.harvard.edu/pph2). In addition, the truncated region at the COOH-terminal is overlapped with the region deduced to be important for dimerization in ABCD1 described above. It is therefore speculated that the mutant ABCD4 in patient 1 is not able to form the proper conformation. In patient 2, the deletion of p.D143_S181 in ABCD4 is supposed to result in lacking TMD3 based on the predicted topology. Consequently, the overall structure of the passageway for the substrate across the membrane that is composed of twelve-transmembrane segments is completely disrupted. In addition, the p.N141K mutation in ABCD4 identified in patients 3 and 4 is predicted to be “damaging” by both PolyPhen-2 and SIFT programs (http://sift.jcvi.org/). The region of AA.141–144 (NPDQ) is conserved among all ABCD transporters.

## 6. Clinical Symptoms of X-ALD and the Development of Therapeutics

There are several X-ALD phenotypes that are categorized based on the onset and severity of the disease: the childhood cerebral form (CCALD), the adolescent cerebral form (AdoCALD), the adult cerebral form (ACALD), adrenomyeloneuropathy (AMN), and Addison's disease alone (Table 1) [54]. The most frequent forms are CCALD and AMN. The CCALD form is associated with inflammation of the cerebral white matter, cerebral demyelination, and adrenocortical insufficiency, with the onset occurring in childhood between 3 and 10 years of age. In contrast, AMN is characterized by a slowly progressive axonopathy in males with an onset of around 20 to 30 years old.

The type of a given ABCD1 mutation, such as missense, nonsense, frameshift, deletion, or insertion, does not predict the disease phenotype. Different clinical phenotypes have even been reported in monozygotic twins [64]. It has been also reported that X-ALD patients with a complete absence of ABCD1 due to large deletions exhibit a milder AMN phenotype [59]. Therefore, modifying influences such as genetic, epigenetic, and environmental factors seem to be important in determining the severity of the disease [59]. Matsukawa et al. have reported SNPs of ABCD1, ABCD2, ABCD3, and ABCD4 genes in X-ALD patients. However, there were no significant associations between these SNPs and phenotypes [65].

At present, there is no effective treatment for preventing the onset or the progression of the neurological pathogenesis that occurs in X-ALD. Allogeneic hematopoietic stem cell transplantation (HSCT) is the only currently available therapeutic procedure, which halts the progress of cerebral demyelination in patients with early stages of CCALD [66]. The mechanism by which HSCT stops demyelination has not yet been made clear. It seems likely that HSCT allows stem cells to penetrate into the brain parenchyma through the brain blood barrier, where the engrafted stem cells fulfill a role by acting as microglia-like cells. However, HSCT is still associated with a higher risk of mortality because of the graft-versus-host reaction and the immunodeficiency induced by myeloablation. Autologous HSC-gene therapy, in which the ABCD1 gene is transduced into hematopoietic stem cell using a lentivirus vector, has been tested as an alternative option. It is an important advantage for CCALD patients without HLA-matched donors. In 2009, Cartier et al. reported that lentiviral-mediated gene therapy of hematopoietic stem cells halted the progression of X-ALD [67]. However, caution is required because of the issue of genotoxicity.

In addition to HSCT-based therapy, pharmacological therapies are still of importance in order to delay both the onset and progression of the disease. Many candidate compounds have been reported for X-ALD therapy to date. The targets of these compounds can be categorized into 4 groups [68]: pharmacological induction of ABCD2 gene expression [69], stimulation of residual peroxisomal VLCFA *β*-oxidation [70], suppression of excess fatty acid elongation [71], and reduction of oxidative stress [72]. However, no promising drug has emerged, so further studies are required for the development of effective treatment.

## Figures and Tables

**Table 1:** Clinical forms of X-ALD.

Phenotype	Ratio (%)	Age of onset
Cerebral ALD		
Childhood cerebral ALD	~38	3–10
Adolescent cerebral ALD	~7	11–21
Adult cerebral ALD	~5	21~
Adrenomyeloneuropathy (AMN)	~50	20–30
Addison's only		
